# Preoperative Glutamine Supplementation in Gastric Cancer—Thrombocyte Phagocytic Activity and Early Postoperative Outcomes

**DOI:** 10.3390/nu15132911

**Published:** 2023-06-27

**Authors:** Zbigniew Kamocki, Joanna Matowicka-Karna, Anna Jurczuk, Anna Milewska, Amanda Niewinski, Konrad Zareba, Boguslaw Kedra

**Affiliations:** 1 Second Department of General and Gastroenterological Surgery, Medical University of Bialystok, M. Sklodowskiej-Curie Street 24a, 15-276 Bialystok, Poland; 2 Department of Clinical Laboratory Diagnostics, Medical University of Białystok, 15-269 Białystok, Poland; joanna.matowicka@umb.edu.pl; 3 Medical University of Bialystok Clinical Hospital, 15-276 Bialystok, Poland; 4 Department of Biostatistics and Medical Informatics, Medical University of Bialystok, 15-089 Bialystok, Poland; 5 English Division, Medical University of Bialystok, 15-089 Bialystok, Poland

**Keywords:** gastric cancer, glutamine, nutritional status, blood platelet phagocytosis, postoperative complications

## Abstract

Background: The aim of this study was to determine the phagocytic activity of thrombocytes in patients with gastric cancer and to assess the effect of oral and parenteral preoperative glutamine-based immunonutrition on nutritional status, thrombocyte phagocytic activity, and early postoperative outcomes. Methods: Patients suffering from invasive gastric cancer had been treated with preoperative immunonutrition with glutamine, and they were compared to patients without nutritional treatment. Nutritional status, percentage of weight loss, and BMI were assessed. Levels of total protein, albumin, cholesterol, triglycerides, platelets, and their phagocytic ability were measured twice. Postsurgical complications were assessed via the Clavien–Dindo classification. Results: Group I consisted of 20 patients with an oral glutamine—10 g daily. Group II had 38 patients who received intravenous glutamine, 1.5 mL per kg body weight of Dipeptiven. Group III consisted of 25 patients who did not receive preoperative immunonutrition. In total, 47% of patients in Group I, 54% of patients in Group II, and 33% of patients in Group III were malnourished. In Group I, the percentage of phagocytizing platelet (%PhP) was 1.1 preoperatively and 1.2 postoperatively. The phagocytic index (PhI) was 1.0 and 1.1. In Group II, %PhP was 1.1 and 1.2 and PhI was 1.0 and 1.1. In Group III, the %PhP was 1.0 and 1.2 and PhI was 1.0 and 1.1. An increase in triglyceride level was observed in both immunonutrition groups. There was a decline in total protein and albumin level in Group II. In Group III, there was a decline in total protein, albumin, and cholesterol level. The total platelet count and PhI were increased in both immunonutrition groups. There was also a rise in %PhP in Group II. In Group III, there was a rise in blood platelet level, %PhP, and PhI. The complication rates were 53% in Group I, 29% in Group II, and 40% in Group III. Conclusions: In invasive gastric cancer, laboratory nutritional parameters are significantly reduced, causing malnutrition in 44.7% of patients. Oral glutamine supplementation inhibited the postoperative decline in protein metabolism parameters; however, this did not affect the reduction in the percentage of postoperative complications. Glutamine used preoperatively significantly reduced the percentage of serious surgical complications, regardless of the way it was supplemented. Patients with invasive gastric cancer have a significant decrease in platelet phagocytic activity. The administered preoperative parenteral nutrition and the surgical procedure itself influenced the improvement of the phagocytic activity of blood platelets. Glutamine did not have this effect, regardless of the route of administration.

## 1. Introduction

Gastric cancer incidence worldwide has declined significantly over the last 30 years. Nevertheless, it still constitutes a significant clinical problem. In 2015, stomach cancer caused 754,000 deaths globally, which makes it the fourth cancer-related cause of mortality [1,2]. Despite progress in diagnostic and therapeutic management, therapy of invasive gastric cancer is associated with a high morbidity and mortality rate, as well as a low quality of life. Postoperative complication rates after total gastrectomy range from 9 to 47.5% and reported mortality rates vary between 1.1 and 10.8% [3,4,5,6]. The most important causes of poor post-operative outcomes are late diagnosis, tumour-related cachexia, and decreased food intake due to anorexia with various secondary causes such as xerostomia, nausea, vomiting, malabsorption, reduced intestinal motility, abdominal pain, chemosensory alteration leading to pre-operative malnutrition in as many as 85% cases [7], and impaired host immune system [8]. Gastric cancer activates a systemic inflammation response syndrome which can vary in severity but impacts all relevant metabolic pathways. Due to this, inflammation induces altered protein turnover, increasing the production of acute phase proteins, and carbohydrate metabolism is impaired due to increased insulin resistance and altered glucose tolerance. The capacity for lipid oxidation is maintained or even increased. Consequently, the loss of mass of skeletal muscles with or without the loss of fat is the main aspect of cancer-associated malnutrition. Muscle protein depletion affects quality of life, physical functioning, and ability to tolerate treatment [9].

**Figure 1:**
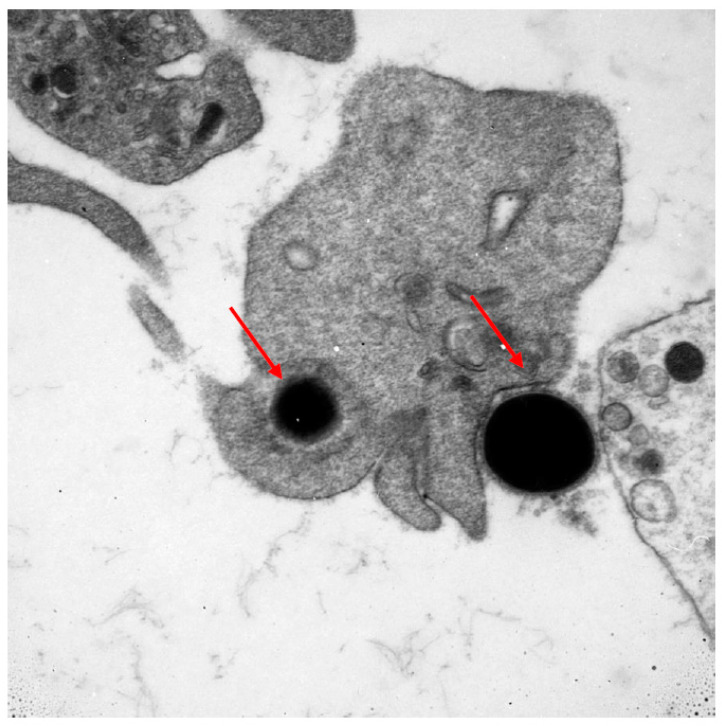
Electron microscope image documenting the phagocytosis of bacteria with electron-dense elementary bodies.

Numerous cells of the immune system are involved in the fight against cancer. Lymphocytes are the first line of defence. The T and B (tumour-infiltrating lymphocytes) subpopulations are well known to, without any geometric modification, kill tumour cells. The NK subpopulation of lymphocytes is also the first line of destruction of cancer cells. Other cells which may potentially play a role in the fight against cancer are thrombocytes. This ability might result from their large number and wide distribution within the circulatory system. Predominantly known for their role as primary mediators of coagulation, this function links them with the occurring inflammatory processes. Platelets, by their biological mediators, can modulate immune response by intracellular crosstalk with leukocytes, lymphocytes, monocytes, and endothelial cells. Thrombocytes play a role in cancer metastases through the transfer of cancer cells and tumour angiogenesis by releasing different factors from their surface, α-granules, and dense granules [10,11,12]. Mustard et al. in 1968 showed that blood platelets have the capacity to phagocytise [13]. Having an ability of chemotaxis and diapedesis, they are able to phagocytise bacteria, viruses, antibody complexes, collagen, and latex particles, working both as single platelets as well as in aggregates [14,15]. Upon activation, thrombocytes change their shape from discoid to an irregular shape with numerous projections. This morphological change is accompanied by the intensification of energetic processes and enhanced protein anabolism inside the activated platelet [16]. In Figure 1, a photo taken with an electron microscope documents the phagocytosis of bacteria with electron-dense elementary bodies. The phagocytic ability of blood platelets in cancer has not been fully determined. 

Our own and other authors’ studies show that phagocytic ability, as well as the bactericidal capacity of blood platelets, is impaired in patients with gastric cancer. The loss of these abilities increases with the clinical advancement of the cancer. The disturbances in the phagocytic ability of thrombocytes may play a role in the pathogenesis of the cancer and may be prognostic. Kamocki et al. demonstrated the loss of phagocytic capacity of blood platelets in inoperable gastric cancer [17]. The platelet function may be partially improved by immunostimulating nutritional therapy. Therefore, there is justified research on immunologically active compounds, such as glutamine, arginine, and ribonucleic acids, which sufficiently improve the host’s immune system. The promising abilities of glutamine did not bring the expected results in clinical practice.

Glutamine, an alpha-amino acid, is one of the main substrates utilised in immunonutrition. It plays a major role as a fuel source for macrophages, lymphocytes, fibroblasts, and enterocytes. Moreover, it provides energy for intestinal epithelial cells and protects the intestinal immune barrier against microbes. Glutamine is a semi-essential amino acid and even though it is endogenously synthetized in physiological conditions, its levels acutely decrease in a catabolic state. Critically ill patients have been shown to have severe glutamine level depletions. Low plasma glutamine levels are related to both the actual and predicted hospital mortality rates [18]. On the other hand, glutamine is essential for cellular proliferation, tumour growth, and cancer cell survival. In 44 patients with resectable gastric cancer, preoperative supplementation of intravenous glutamine with a fish-oil-based fat emulsion partially improved the phagocytic function of thrombocytes [17]. However, the role of glutamine supplementation in clinical practice is still controversial [19,20]. 

The potential methods of optimising cancer treatment outcomes include the use of immunogenic therapies to re-establish host anti-tumour immune response. Perioperative immunonutrition reduces the risk of surgical site infection and significantly decreases white blood cell count and C-reactive protein levels. Furthermore, patients receiving perioperative immunonutrition or early enteral nutrition were shown to have shorter in-hospital length of stay [21]. Oral solutions of arginine, administered preoperatively along with adjunct nutritional therapy in advanced gastric cancer patients with retained gastrointestinal passage, significantly increase the fraction of phagocytising platelets and improve the phagocytic index of thrombocytes [19].

ESPEN (European Society for Clinical Nutrition and Metabolism) recommends the use of perioperative oral and/or enteral immunonutrition in surgical patients with upper gastrointestinal cancer [22]. 

Aim: The aim of this study was to determine the impact of preoperative glutamine-based immunonutrition on the phagocytic activity of blood platelets in patients with gastric cancer. The effects of both intravenous infusion of glutamine and oral supplementation were assessed.

## 2. Methods

### 2.1. Study Population

Patients with invasive gastric cancer were enrolled in this prospective clinical trial. The only inclusion criterion was operable gastric cancer, regardless of the TNM (Classification of Malignant Tumours) stage. Exclusion criteria included inoperable gastric cancer and thrombocytopenia. 

On admission to the hospital, each patient was evaluated for nutritional status using SGA (Subjective Global Assessment) score. SGA identifies patients who may benefit from nutritional therapy by assessing nutrient intake, weight loss, symptoms, functional capacity/protein calorie malnutrition, metabolic requirement, body composition (fat and muscle wasting), oedema, and ascites. The percentage of unintentional weight loss and BMI (body mass index) were then calculated. Blood samples were obtained twice. On hospital admission, each patient had total protein, albumin, total cholesterol, triglyceride, platelet count, and phagocytic activity assessed. Laboratory tests and phagocytic activity were reassessed 12 days postoperatively. 

### 2.2. Randomization 

Patients were randomized into three groups based on the route of glutamine supplementation they would receive. The period of glutamine supplementation lasted for 8 to 14 days prior to gastrectomy (mean 12 days) in Group I, which received oral glutamine, and Group II, which received intravenous glutamine. Group III received no glutamine. The only condition for qualification to Group I was unimpaired gastric emptying. 

### 2.3. Preoperatively

All three groups received a regular hospital diet supplemented with a three-chamber SmofKabiven Peripheral parenteral feeding bag (1447 mls, Fresenius Kabi AB, GTIN number 05909990723041\n05909990754809\n05909990722976\n05909990754823\n) fortified with one ampule of vitamins (daily doses of all water- and fat-soluble vitamins, with exception of vitamin K, Cernevit, Baxter Poland, R/6576). Patients in Group I also received an oral glutamine solution twice a day—10 g of L-glutamine (Resource Glutamin^®^ (Vevey, Switzerland), Nestle Health Science, catalogue number 12095417). Group II received the same peripheral feeding bag and vitamins as Group I, as well as a 100 mls intravenous solution of glutamine (N (2)-L-alanyl-L-glutamine dipeptide (20 g N (2)-L-alanyl-L-glutamine, 8.2 g L-alanyl and 13.46 g L-glutamine)) with 0.2 g/mL of the medicinal product administered (Dipeptiven, Fresenius Kabi Zealand Limited, R/7330, 1.5 mL per kg body weight according to the manufacturer’s recommendation). Group III received no glutamine. 

### 2.4. Postoperative

In all patients, early enteral nutrition with a polymeric, normocaloric, and low-fat diet was started 20 h after surgery. In the case of type 2 diabetes mellitus, patients received a special enteral formula normalising glycaemia. Additionally, parenteral nutrition without glutamine (SmofKabiven Peripheral three-chamber bag) was supplemented for 5–6 days after surgery (mean 5.4 days) in all groups. Oral feeding started in the 5th day after surgery. Low-molecular-weight heparin was used in the prevention of blood clots in each patient postoperatively. 

### 2.5. Data Collection

#### 2.5.1. Surgery

Open total gastrectomy included stomach resection with excision of regional lymph nodes in the D2 territory. Two methods of alimentary tract reconstruction were performed: Roux-en-Y and double tract reconstruction. In the latter method, the Roux-en-Y loop was additionally connected to the duodenum, approximately 35 cm below the esophageojejunal anastomoses. A nasojejunal feeding tube was placed intraoperatively into the alimentary limb, 15 cm below the jejunojejunal anastomosis.

Thirty-day mortality rate and complications were recorded and stratified according to the Clavien–Dindo classification [23].

#### 2.5.2. Imaging Technique and Imaging Analysis

The phagocytic activity of blood platelets was assessed at 8–14 days prior to surgery and on day 12 postoperatively. The activity was determined against Staphylococcus aureus ATCC 653P bacterial strain, according to Mantur et al.’s method [24]. Phagocytic activity and phagocytic index were expressed as the percentage of phagocytizing platelets and the phagocytic index. To determinate blood platelet activity, the bacterial suspension and platelet-rich plasma were incubated at 370 degrees Celsius for 6 min and then mixed at 75 g. After incubation, smears were made and stained with the Pappenheim method for 1 h using Giemsa reagent. The stained preparations were evaluated under a light microscope at ×1400 magnification. The percentage of phagocytizing platelets was described as the percentage of phagocytizing platelets per 1000 other cells in the preparation. The phagocytic index was determined using 100 phagocytizing platelets. The index was calculated as the mean number of phagocytized bacteria per single platelet according to the following formula: phagocytic index = number of phagocytized bacteria/numbers of phagocytizing platelets. 

#### 2.5.3. Statistical Analysis

Normally distributed data are expressed as medians and standard deviations.

The results were analysed using the Statistica 13.1 program, *p* < 0.05, using the Wilcoxan signed-rank test.

#### 2.5.4. Trial Registration and Ethical Approval

All procedures followed were in accordance with the ethical standards of the institutional and national committee on human experimentation and the Helsinki Declaration. Written informed consent was obtained from all patients. 

This study was approved by the Bioethical Commission at the Medical University of Bialystok, no.: R-I-022/149/2007. This research was funded by the Medical University of Bialystok (research number: N/ST/ZB/17/001/1137). This trial was registered with Clinicaltrials.gov (NCT01704664).

## 3. Results

Eighty-three patients with resectable invasive gastric cancer were recruited from the Department of General and Gastroenterological Surgery between 2012 and 2017. Overall, there were 25 females (30%) and 58 males (70%) aged 27 to 84 (63.5 ± 14). 

**Figure 2:**
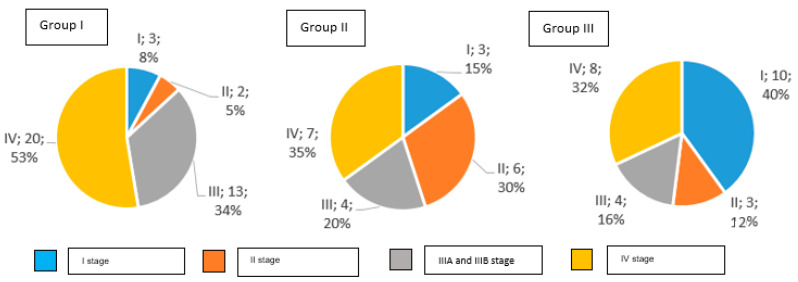
Clinical stages of gastric cancer in examined groups of patients according to AJCC TNM classification (number and percentage of patients).

The distribution of clinical stages in particular groups is presented in Figure 2.

In Group I, 92% of study participants were diagnosed with an invasive gastric cancer, while Group II had 85%, and Group III had 60%. Early gastric cancer was detected in 8% of patients in Group I, in 15% of Group II, and in 40% of Group III.

### 3.1. Group I—Oral Glutamine

Group I included 20 patients (7 women, 13 men) aged from 34 to 82 years (65.2 ± 11.9). Unintentional weight loss of more than 10% was demonstrated in seven patients (35%). The SGA scale showed malnutrition in 47% of patients. The percentage of reported total body weight preoperatively ranged from 10 to 25% (15.3% ± 6). The body mass index ranged from 16 to 28 kg/m^2^ (26.3 ± 7.29). 

A decreased protein level was found in nine patients, and a low albumin level was found in sixteen patients. 

Postoperatively, the level of total protein and albumin did not change significantly. The levels of cholesterol and triglycerides improved postoperatively. 

The preoperative and postoperative serum levels of total protein, albumin, cholesterol, and triglyceride in patients of Group I are shown in Table 1. 

### 3.2. Group II—Intravenous Glutamine 

Group II consisted of 38 patients (8 women, 30 men) aged from 45 to 84 years (66.9 + 10.4). Unintentional weight loss of more than 10% was demonstrated in fifteen patients (50%). Malnutrition was observed in 54% patients. The percentage of body weight lost ranged from 10 to 30% (15.7% + 6.1). The body mass index ranged from 18 to 40 (24 + 4.2). A low protein level was found in 17 patients, and a low albumin level was found in 19 patients.

Postoperatively, the levels of albumin were significantly lower, but there was no change in the total protein. An increase in the minimum and decrease in the maximum values of cholesterol and triglycerides was observed, which signifies the improvement of lipid metabolism in these patients. 

The preoperative and postoperative serum levels of total protein, albumin, cholesterol, and triglyceride in patients of Group II are shown in Table 2. 

### 3.3. Group III—Nutrition without Glutamine

Group III consisted of 25 patients (10 women, 15 men) aged from 27 to 83 years (63.5 + 13.8). Unintentional weight loss above 10% was demonstrated in four patients (16%). In 33% of patients, malnutrition was observed. The percentage of body weight lost ranged from 10 to 15% (12.5% + 2.9). The body mass index was from 19 to 35 (25.2 + 4.0). 

A low protein level was found in five patients, and a low albumin level was found in eight patients.

Postoperatively, the level of albumin decreased significantly without any changes in the level of total protein. The cholesterol and triglyceride levels significantly improved postoperatively. The observed changes in the cholesterol level indicate its increased metabolism in the postoperative period. There was a significant increase in the triglyceride levels postoperatively. 

The preoperative and postoperative serum levels of total protein, albumin, cholesterol, and triglyceride in patients of Group III are shown in Table 3.

### 3.4. Blood Platelets and Their Phagocytic Activity 

The preoperative and postoperative blood platelet count, phagocytic activity, and phagocytic index of patients in Group I are shown in Table 4. 

A relevant increase in the total count of platelets and index was noticed when comparing the preoperative count to the postoperative count. The phagocytic index was statistically increased, without any changes in the phagocytic activity of blood platelets. 

In Group II and Group III, there was a statistical increase in the postoperative number of total platelet count, phagocytic activity, and phagocytic index. The preoperative and postoperative total count of platelets, phagocytic activity, and phagocytic index are shown in Table 5 and Table 6. 

### 3.5. Complications

In Group I, the overall postoperative complication rate was 53%, with major complications observed in 33% of patients. The 30-day mortality was 12%. 

In Group II, the overall postoperative complication rate was 29%, with major complications seen in 40% of patients. The 30-day mortality was 6%.

In Group III, the overall postoperative complication rate was 40%, with major complications in 83% of patients. The 30-day mortality was 6%. 

In Table 7, the surgical complications observed in the examined groups were analysed according to the Clavien–Dindo classification. The observed complication rate did not prove to be statistically significant when analysing the three groups. 

## 4. Discussion

Different surgical techniques have various local and general imbroglios [6]. One of the most important problems in the treatment of patients suffering from stomach cancer is their proper preoperative preparation. The malignancy process is associated with malnutrition, as well as with the impairment of the host immune defence. Furthermore, the increase in energy–protein expenditure during the surgery increases malnutrition and exacerbates the risk of perioperative complications. In multivariate analysis, malnutrition was an independent factor for more postoperative infections. The excessive loss of protein and energy observed in patients with advanced cancer is associated with increased morbidity, poor response to chemotherapy, and a shorter survival time [25]. Li et al. reported cachexia in 73.3% of patients with gastric cancer [26]. Prolonged malnutrition during early life may increase the risk of stomach cancer mortality in later life; hence, the nutritional status needs to be optimised in the preoperative period [27]. Fukuda et al. analysed 800 gastric cancer patients who had undergone a gastrectomy. They classified 19% of patients as malnourished. In our work, the assessment of nutritional status was based on the SGA classification, and biochemical analysis showed malnutrition in 47% of patients in Group I, 54% of patients in Group II, and 33% of patients in Group III. Unintentional weight loss of more than 10% of their body weight in the last 6 months was found in 34% of patients. The mean unintentional weight loss was 13.7 kg. In relation to the work written by Yoon, unintentional weight loss in patients presenting with gastric cancer was implicated in lowered survival rates and quality of life [28]. Unintentional weight loss in gastric cancer patients, especially in stage 3, has been recognized as an independent prognostic factor [29].

The current ESPEN guidelines recommend about a 10–14 day period of feeding with immunologically active compounds [22]. Well-managed preoperative nutritional support decreased the incidence of postoperative surgical site infections [30]. 

Liu et al. found that preoperative BMI was positively correlated with albumin and triglyceride levels, and preoperative albumin levels were positively correlated with triglycerides. Therefore, serum albumin level is not only a window into the patients’ nutritional status but is also a useful factor for predicting prognosis [31]. Poor survival was observed in gastric cancer patients with lower levels of BMI, albumin, and triglyceride [32]. Because low levels of serum albumin are associated with poor outcomes in cancer patients, they can be used as an independent indicator when assessing the need for aggressive nutritional intervention [33]. 

Glutamine-supplemented perioperative nutrition has been investigated in patients with a variety of diseases, but the effects have not been conclusively established [34]. In our study, two different models of preoperative immunonutrition with glutamine were analysed and compared to a group of patients fed parenterally without glutamine. Nowadays, the new model of gastric cancer treatment based on preoperative radiotherapy and chemotherapy forced us to reduce the number of examined patients, but the achieved results allow us to make conclusions. 

The laboratory data for protein and lipid metabolism were observed to be similar in all three groups. Preoperative nutrition allowed the protein to remain unchanged postoperatively, but the level of albumin significantly decreased in all groups. In addition, triglycerides increased in all three groups. Cholesterol values also significantly decreased in Groups II and III but increased in the group receiving oral glutamine-enriched immunonutrition. In the literature, it is said that glutamine used during preoperative immunonutrition helped to sustain higher albumin levels whilst, on the other hand, a decrease in total protein level was recorded. Tue et al. have also observed an improvement in postoperative cumulative nitrogen balance with perioperative parenteral nutrition supplemented with glutamine in patients undergoing abdominal surgery [35]. One explanation for this protein metabolism may be a reduced production of proinflammatory cytokines. In a previous study by our group, we found elevated levels of interleukin 6 (IL-6) in gastric cancer patients which correlated negatively with the disease stage. Its values were highest in patients with early gastric cancer [36]. Glutamine-enriched nutrition support in surgical patients remains controversial. A meta-analysis with thirteen randomized controlled trials showed an improved immune function, a reduced incidence of infectious complications, and a shortened length of hospital stay after this type of nutrition [37]. Jiang et al. noted that alanyl-glutamine-supplemented parenteral nutrition is clinically safe with better nitrogen balance and maintained intestinal permeability in postoperative patients when compared with patients who received isonitrogenous and isocaloric parenteral nutrition [38]. The highest percentage of postoperative complications was found in Group I, and it was almost twice as high compared to patients in Group II. Perhaps this is due to the different numbers of patients. However, an interesting result is a similar percentage of severe complications in both groups with preoperative immunonutrition. Major postoperative complications were almost three times more common in patients without preoperative immunonutrition. Similarly, the different number of patients in each group influenced the mortality percent rate. The highest percent of mortality was noted as 12% of patients in Group I, compared to 6% in Group II and 6% in Group III. Giannotti et al. did not report better surgery outcomes with parenteral glutamine supplementation in well-nourished patients with gastrointestinal cancer [39].

A high number and wide distribution within the circulatory system make thrombocytes an important component of the immune system, but their role in the immune response is not yet fully understood. Platelet granules contain peroxidase, acid phosphatase, cationic proteins, and proteolytic enzymes. These substances represent high activity for bacterial phagocytosis. Furthermore, platelets exert cytotoxic effects on cancer cells by adhering to them via antigenic determinants. Then, characteristic structural changes, such as Golgi apparatus hypertrophy, an increase in secretory granulations, and their displacement towards a contact zone with a neoplastic cell, are observed. Platelet cytotoxicity ensues from their ability to produce and release lytic mediators [40]. Additionally, platelets play an important role in tumour metastasis. Platelet-delivered proteolytic enzymes facilitate the release and migration of tumour cells across the vessel wall [41]. Furthermore, activated platelets release substances which increase vascular permeability, factors stimulating myocyte proliferation, platelet-activating factor, prostaglandins, histamine, and serotonin. The substances above facilitate an implantation and growth of metastatic tumours [42]. In our last studies, we found that the fraction of phagocytizing platelets and their phagocytic index in gastric cancer patients was markedly impaired as compared to healthy individuals [43]. The current work confirms impaired phagocytic activity in patients with gastric cancer. A decreased phagocytic activity can influence inflammatory processes as well as cancer growth [36]. In this study, the impact of immunonutrition on the phagocytic activity of platelets was evaluated. Glutamine is an essential amino acid for fast-dividing immune cells, epithelial cells of the gastrointestinal tract, fibroblasts, and reticulocytes. It is a precursor of protein and nucleotide synthesis; it is also involved in hepatic gluconeogenesis and glutathione synthesis. Its high concentration was found in the intestinal mucosa cells. Glutamine and glutamate are amino acids responsible for the transport of nitrogen and the detoxification of ammonia. The consequence of the inhibition of glutathione synthesis is mucosal destruction, diarrhoea, and growth inhibition. Parenteral supplementation of glutamine in rats demonstrated its protective role against bacterial translocation [44]. The impact of glutamine on blood platelet phagocytic activity is unknown. 

In comparison to our previous study, all groups showed severe impairment of blood platelet phagocytic activity. The percentage of phagocytizing platelets and their phagocytic index in all patients was observed to have declined. Postoperatively, the percentage of blood platelets and number of phagocytizing platelets was increased in all groups, and phagocytic index was significantly increased, except in Group I. These data confirm the improvement in the postoperative activity of phagocytic platelets depending on the performed surgery with preoperative parenteral nutrition. The addition of glutamine, orally or parenterally, does not have an influence on the platelet activity. It is important to pay attention to the fact that surgical processes stimulate the production of new platelets, which are characterized by more biological activity. Further testing is required for the assessment of whether glutamine is a factor in the improvement of the phagocytic ability of platelets.

The authors had previously reported partial improvement in the thrombocyte phagocytic activity in gastric cancer patients as a result of perioperative immunonutrition enriched with glutamine and ω-3 fatty acids both in local disease and in peritoneal dissemination [43]. This is the first study which analyses blood platelet phagocytic activity in gastric cancer patients receiving an enteral glutamine diet preoperatively. Additionally, data from patients with preoperative nutrition which was not enriched with glutamine were assessed.

## 5. Conclusions

In invasive gastric cancer, laboratory nutritional parameters are significantly reduced, causing malnutrition in 44.7% of patients. Oral glutamine supplementation used in preoperative nutritional therapy inhibited the postoperative decline in protein metabolism parameters; however, this did not affect the reduction in the percentage of postoperative complications. Glutamine used in preoperative immunonutrition significantly reduced the percentage of serious surgical complications, regardless of the way it was supplemented. The administered preoperative parenteral nutrition and the surgical procedure itself influenced the improvement of the phagocytic activity of blood platelets. Glutamine did not have this effect, regardless of the route of administration.

## Figures and Tables

**Table 1:** Preoperative and postoperative serum levels of total protein, albumin, cholesterol, and triglyceride in patients of Group I.

	Preoperative				Postoperative				(*p* Wilcoxon)
	Min	Max	Median	SD	Min	Max	Median	SD	
Total protein (g/dL)	4.60	7.50	6.0	2.05	4.80	7.40	5.8	1.84	0.8445
Albumin (g/dL)	2.57	4.12	3.23	1.1	2.20	3.70	2.85	1.06	0.2132
Cholesterol (mg/dL)	102	232	150	38.19	84	195	156	31.11	0.0211
Triglyceride (mg/dL)	34	351	92	9.9	57	218	110	18.38	0.0015

**Table 2:** Preoperative and postoperative serum levels of total protein, albumin, cholesterol, and triglyceride in patients of Group II.

	Preoperative				Postoperative				(*p* Wilcoxon)
	Min	Max	Median	SD	Min	Max	Median	SD	
Total protein (g/dL)	4.80	7.60	5.9	1.98	4.90	7.10	5.8	1.56	0.1358
Albumin (g/dL)	1.86	4.31	3.4	1.73	1.98	3.83	2.82	1.31	0.0000
Cholesterol (mg/dL)	74	264	159	2.12	78	235	143	4.24	0.0000
Triglyceride (mg/dL)	37	195	103	29.70	51	188	110	14.14	0.0000

**Table 3:** Preoperative and postoperative serum levels of total protein, albumin, cholesterol, and triglyceride in patients of Group III.

	Preoperative				Postoperative				(*p* Wilcoxon)
	Min	Max	Median	SD	Min	Max	Median	SD	
Total protein (g/dL)	5.30	8.0	5.9	0.6	5.0	7.0	5.8	0.6	0.3463
Albumin (g/dL)	1.9	4.0	3.4	0.5	2.0	4.0	2.9	0.5	0.0000
Cholesterol (mg/dL)	79.0	264.0	165.0	43.5	78.0	212.0	142.0	33.5	0.0000
Triglyceride (mg/dL)	37.0	175.0	102	37.4	51.0	188.0	109.0	30.8	0.0000

**Table 4:** The number of blood platelets, the number of phagocytizing platelets, and the phagocytic index in Group I before and after surgery.

	Preoperative				Postoperative				(*p* Mann-Whitney)
	Min	Max	Median	SD	Min	Max	Median	SD	
Platelets	110,000	450,000	268,000	24,041,631	265,000	1,093,000	656,000	58,548,441	0.0034
Phagocytizing platelets	1.0	1.2	1.1	0.14	1.0	1.3	1.1	27.58	0.0051
Phagocytic index	1.0	1.1	1	32.53	1	1.2	1.1	27.58	0.337

**Table 5:** The number of blood platelets, the number of phagocytizing platelets, and the phagocytic index in Group II before and after surgery.

	Preoperative				Postoperative				(*p* Mann-Whitney)
	Min	Max	Median	SD	Min	Max	Median	SD	
Platelets	132,000	532,000	239,000	93,289,305	180,000	1,152,000	709,000	12,723,538	0.0000
Phagocytizing platelets	1	1.3	1.1	0.21	1	1.4	1.2	0.07	0.0002
Phagocytic index	0.8	1.2	1	0.8	0.8	1.3	1.1	0.35	0.0003

**Table 6:** The number of blood platelets, the number of phagocytizing platelets, and the phagocytic index in Group III before and after surgery.

	Preoperative				Postoperative				(*p* Mann-Whitney)
	Min	Max	Median	SD	Min	Max	Median	SD	
Platelets	1,320,000	532,000	2,270,000	1,043,789	1,800,000	1,152,000	7,090,000	2,880,189	0.0001
Phagocytizing platelets	1.0	1.3	1.1	0.1	1.0	1.4	1.2	0.1	0.0004
Phagocytic index	0.8	1.2	1.0	0.1	0.8	1.3	1.1	0.1	0.0004

**Table 7:** Postoperative complications in all groups according to the Clavien–Dindo classification (14).

Grade	Group I	Group II	Group III
I	1	2	
II	7	5	1
IIIa	-	1	2
IIIb	-	1	2
IVa	1	-	1
IVb	-	1	2
V	2	2	2

## Data Availability

The raw data generated and analysed in the current study are not publicly available due to the appropriate protection of patient personal information but are available from the corresponding author on reasonable request.

## Abbreviations

SGA	Subjective Global Assessment
BMI	Body mass index
TNM	Classification of Malignant Tumours
PhIT	Phagocytic index
PhT%	Percentage of phagocytizing thrombocytes
ESPEN	European Society for Clinical Nutrition and Metabolism
